# Interference of Continuous Renal Replacement Therapy on PiCCO Hemodynamic Monitoring: A Case Report

**DOI:** 10.1002/ccr3.71057

**Published:** 2025-10-17

**Authors:** Xiaohua Lin, Zhijun Suo, Xuan Liu, Haigang Zhang, Yunsheng Yuan

**Affiliations:** ^1^ Department of Critical Care Medicine Shenzhen Nanshan People's Hospital Shenzhen China

**Keywords:** case report, catheter, continuous renal replacement therapy, hemodynamic measurements, pulse index continuous cardiac output, transpulmonary thermodilution

## Abstract

The proximity of the central venous and dialysis catheters during continuous renal replacement therapy can interfere with Pulse Index Continuous Cardiac Output hemodynamic measurements. Adjusting catheter placement or temporarily pausing continuous renal replacement therapy during measurement is essential to ensure accurate hemodynamic assessment in critically ill patients.

**Summary**

- Accurate hemodynamic assessment using PiCCO in patients undergoing continuous renal replacement therapy is critical; interference may occur due to catheter proximity.
- Temporary cessation of CRRT during PiCCO calibration can enhance measurement accuracy and guide better clinical decisions.

## 1 Introduction

Pulse index Continuous Cardiac Output (PiCCO) monitoring employs transpulmonary thermodilution (TPTD) for initial calibration and provides real‐time hemodynamic parameters essential for goal‐directed management in critically ill patients [1]. The calibration process involves injecting 15–20 mL of cold saline via a central venous catheter. However, in patients undergoing continuous renal replacement therapy (CRRT), the proximity of the dialysis catheter to the central venous catheter can lead to significant inaccuracies in hemodynamic measurements.

Several studies have highlighted the challenges of accurate hemodynamic monitoring in patients receiving CRRT. Schmidt et al. [2] demonstrated that renal replacement therapy can significantly affect TPTD measurements, leading to underestimation of cardiac index and overestimation of extravascular lung water. Herner et al. [3] found that the cardiac function index derived from femoral indicator injection for TPTD is significantly lower when CRRT is active, further highlighting the potential for interference. Litton and Morgan [4], in their review of PiCCO monitoring, emphasized the importance of understanding the limitations of the technique, especially in the context of concurrent therapies. Therefore, this potential interference warrants careful consideration.

We report a case of a patient undergoing CRRT where initial PiCCO measurements conflicted with echocardiographic findings, highlighting the importance of awareness and troubleshooting in such situations.

## 2 Case History/Examination

A 62‐year‐old male patient with severe acute myocardial infarction and mitral regurgitation underwent coronary artery bypass grafting, mechanical mitral valve replacement, and tricuspid valvuloplasty. In the postoperative period, he developed colonic necrosis and subsequent septic shock with acute kidney injury, necessitating transfer to our intensive care unit (ICU) for advanced management. At the time of transfer, he required continuous infusions of norepinephrine (0.4 μg/kg/min) and epinephrine (0.1 μg/kg/min), administered through a central venous catheter, to maintain adequate blood pressure.

**FIGURE 1:**
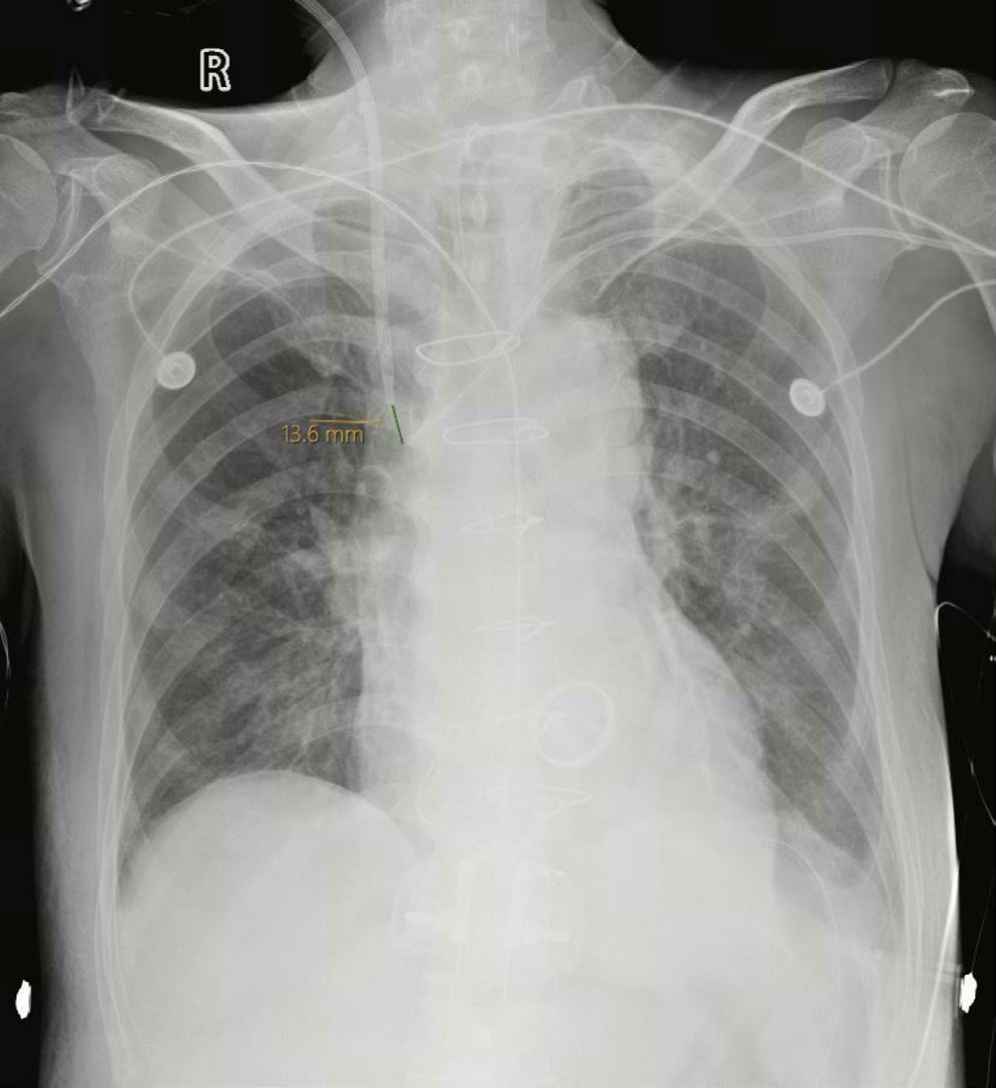
Chest radiograph showing the positions of the central venous and dialysis catheters.

Invasive hemodynamic monitoring was initiated using the PiCCO2 system (Pulsion Medical Systems, Munich, Germany). To facilitate continuous venovenous hemodiafiltration (CVVH‐DF), a 13.5 Fr Niagara double‐lumen hemodialysis catheter (Bard, Murray Hill, NJ, USA) was inserted into the right internal jugular vein. A 7‐Fr dual‐lumen central venous catheter (Bioptimal, Jalan Tukang, Singapore) was inserted into the left subclavian vein, with its location confirmed by bedside chest radiography, demonstrating a tip distance of 13.6 mm (Figure 1). A 4‐Fr PiCCO arterial thermistor was inserted into the femoral artery, and after the patient's body surface area (BSA) was 1.63 m^2^, PiCCO2 calibration was performed with a rapid injection of 15 mL cold saline. Moreover, pulsed‐wave Doppler assessment of the left ventricular outflow tract Velocity Time Integral (VTI) revealed an adequate stroke volume of 18 cm, and there was no evidence of diastolic dysfunction or significant pulmonary hypertension.

## 3 Differential Diagnosis

The differential diagnosis for the hemodynamic instability included septic shock due to colonic necrosis, cardiogenic shock secondary to myocardial infarction, and the effects of fluid overload and renal failure associated with CRRT.

## 4 Conclusion and Results (Outcome and Follow‐Up)

**FIGURE 2:**
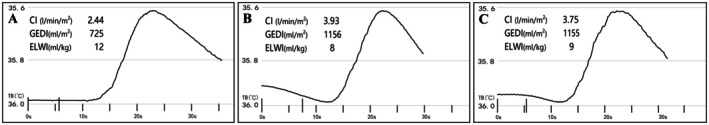
Thermodilution curve (A) Initial thermodilution curve during concurrent CRRT, displaying an extended downslope. (B) Thermodilution curve after stopping CRRT, showing normalized upslope and downslope. (C) Repeat measurement of thermodilution curves without CRRT interference.

The initial thermodilution curve for low cardiac output and hypovolemic state (Figure 2A), these results were inconsistent with the systolic function observed on echocardiography. Suspecting potential interference from the CRRT circuit, we temporarily paused the CRRT blood pump (blood flow at 120 mL/min) and recalibrated the PiCCO2 system. The new measurements showed that the hemodynamic parameters correlated well with the echocardiograms (Figure 2B,C). The cardiac output was 3.75 L/min/m^2^ and the central venous pressure was 12 mmHg, indicating the patient was not hypovolemic. The initial thermodilution curve showed an extended slope compared to the curve obtained after stopping CRRT. This increase in area under the curve (AUC) leads to errors in hemodynamic measurements by the PiCCO2 monitor, resulting in an underestimation of cardiac output.

The antimicrobial regimen of cefepime and metronidazole was continued at the same dosages for a total of 14 days, targeting the identified source of infection related to colonic necrosis. However, despite our best efforts, the patient ultimately succumbed to septic multi‐organ failure 30 days after the initial cardiac surgery.

## 5 Discussion

This case report illustrates a significant clinical challenge: the potential for interference between CRRT and TPTD measurements obtained via the PiCCO2 system, leading to inaccurate hemodynamic assessments. This case is unusual because it highlights the importance of catheter placement for better interpretation and validation with bedside echo; this is not a common practice as many clinicians tend to believe the value obtained from the PiCCO2 device without consideration of other factors.

The pathophysiology of acute kidney injury (AKI) in critically ill patients is complex, often stemming from a combination of factors, including sepsis, ischemia–reperfusion injury, and nephrotoxic medications [5, 6]. The decision to initiate CRRT in our patient was based on established criteria, including oliguria, fluid overload refractory to diuretics, and electrolyte imbalances, in the context of his overall hemodynamic instability.

While the influence of CRRT on TPTD measurements has been previously investigated, this case underscores the importance of vigilant monitoring and integration of clinical data in interpreting PiCCO2 results. Martínez‐Simón et al. [7] described a similar case where CRRT interfered with PiCCO2 measurements, leading to an underestimation of cardiac output. Similarly, Herner et al. [3] demonstrated that the cardiac function index derived from femoral indicator injection for TPTD is significantly lower when CRRT is active. These findings are consistent with our observation that the proximity of the central venous and dialysis catheters can create turbulence and alter the thermodilution curve, leading to inaccurate measurements. In contrast, Dufour et al. [8] found that CRRT with high blood pump flow does not significantly alter TPTD measurements. However, in this study, the flow was lower and therefore a large effect may not have been apparent. Our approach involved temporarily suspending CRRT during PiCCO2 calibration, a strategy that effectively mitigated the interference and allowed for accurate hemodynamic assessment.

## Author Contributions

**Xiaohua Lin:** conceptualization, data curation, methodology, validation, writing – original draft. **Zhijun Suo:** conceptualization, formal analysis, investigation, methodology. **Xuan Liu:** data curation, investigation, methodology, validation. **Haigang Zhang:** conceptualization, methodology, supervision, validation. **Yunsheng Yuan:** methodology, project administration, validation, writing – review and editing.

## Consent

Written informed consent was obtained from the patient for the publication of this case report and any accompanying images. This patient provided oral and signed written consent to use his clinical materials in this study.

## Conflicts of Interest

The authors declare no conflicts of interest.

## Data Availability

Study findings and associated data can be made available by the corresponding author following a reasonable request.
